# Plausible Functional Diagnostics by Rational Echocardiography in the Assessment of Valvular Heart Disease - Role of Quantitative Echocardiography in the Assessment of Mitral Regurgitation

**DOI:** 10.3389/fcvm.2022.819915

**Published:** 2022-03-31

**Authors:** Andreas Hagendorff, Stephan Stöbe

**Affiliations:** Leipzig University Hospital, Department of Cardiology, Leipzig, Germany

**Keywords:** echocardiography, valvular heart disease (VHD), mitral regurgitation, plausibility, hemodynamic

## Abstract

The echocardiographic assessment of valvular heart diseases is the basic analysis of valvular defects next to clinical investigation and stethoscopy. Severity of mitral regurgitation (MR) is usually estimated by an integrated approach using semi quantitative parameters and is still one of the biggest challenges of echocardiography. Quantitative echocardiographic analysis of MR severity often fails to describe comprehensible hemodynamic conditions. However, comprehensive echocardiography based on standardized image acquisition and proper image quality is required to properly assess hemodynamic parameter comparable to cardiac magnetic resonance tomography. This review focuses on the uncertainty of MR severity assessed by echocardiography in recent trials of interventional MR treatment. In addition, the necessity to provide plausible echocardiographic data for individual decision making is highlighted. In conclusion, plausible functional diagnostics by rational echocardiography is a prerequisite in patients with valvular heart diseases.

## Introduction

Echocardiography is usually the primary diagnostic procedure in suspected valvular heart disease (VHD) (1–4). Clarifying the etiology of VHD is the first step of echocardiographic diagnostic. The grading of the severity of the valvular defects is the second target of a comprehensive functional assessment by echocardiography (5, 6). Conventional echocardiographic modalities of transthoracic two-dimensional echocardiography (2D-TTE) to analyze VHD encompass M-Mode, 2D-imaging, color-coded, pulsed wave (pw), and continuous wave (cw) Doppler echocardiography (7). These techniques are extended by tissue Doppler imaging (TDI), speckle tracking echocardiography (STE), multiplane and multidimensional (3D-) echocardiography and advanced approaches such as transesophageal echocardiography (TEE), contrast and stress echocardiography (8). All these modalities can be useful for a proper assessment of valvular defects and their hemodynamic consequences (5, 6). The necessity of a quantitative comprehensive echocardiographic approach to analyze the pathophysiological sequelae of valvular defects is emphasized by using the example of mitral regurgitation (MR). A verifiable quantitative assessment of hemodynamics enables a reliable interpretation of individual results. In consequence, a plausibility check of the quantitative results should be performed prior to individual decision making.

## The Main Problem: The Understanding of Hemodynamics

The grading of the severity of MR by echocardiography should serve as an example to illustrate the main methodological problems. Recent trials provide prognostic data of transcatheter mitral valve repair (*TMVR*)—mainly by transcatheter edge-to-edge repair (TEER)—in functional or secondary MR (FMR) based on the characterization of FMR severity by echocardiography (9–11). The discussion about different results leads to the proposal of a new conceptual framework by creating the terms “proportionate” and “disproportionate” FMR (12–14). In the original paper the authors argued “According to the Gorlin hydraulic orifice equation, patients with heart failure (HF), an LV ejection fraction of 30%, a LVEDV of 220–250 ml, and a regurgitant fraction of 50% would be expected to have an EROA of ≈0.3 cm^2^ independent of specific tethering abnormalities of the mitral valve (MV) leaflets.” and “In contrast, patients with EROA of 0.3–0.4 cm^2^ but a LVEDV of only 160–200 ml exhibit degrees of MR that are disproportionately higher than predicted by LVEDV. These patients appear to preferentially benefit from interventions directed at the mitral valve.” (12). Of course, this is a lot of information in two citations. However, it might be allowed to focus on the calculated hemodynamics of both scenarios despite the Gorlin hydraulic orifice equation is based on the prerequisite of a stable circular—non-physiological—orifice area.

A left ventricle with a left ventricular (LV) end diastolic volume (LVEDV) of 250 ml and a LV ejection fraction (LVEF) of 30% has a total LV stroke volume (LVSV_tot_) of 75 ml. In the presence of a regurgitant fraction (RF) of 50% the effective stroke volume (LVSV_eff_) is 37.5 ml.

In case of a body surface area (BSA) of 1.9 m^2^ (=BSA of a normal adult), a normal cardiac index (CI) is in the range from at least 2 to 2.2 l/min/m^2^, which results in a cardiac output (CO) of at least 3.8 l/min. There are no data about the CI-range in hemodynamically stable FMR patients. However, it can be assumed that the lowest CI in FMR patients might be ≤2 l/min/m^2^, presumably in the range from 1.7 to 2.0 l/min/m^2^.

If LVSV_eff_ is 37.5 ml, a heart rate of 86/min will be necessary to ensure a CI of 1.7 l/min/m^2^ (1700 ml x 1.9 m^2^/37.5 ml). Subsequently, a heart rate of 101/min will be necessary to ensure a CI of 2.0 l/min/m^2^ (2000 ml x 1.9 m^2^/37.5 ml). In contrast to these calculated heart rates the heart rates of HF patients with optimal medical treatment (OMT) are usually in ranges of 55–60/min.

According to the definition of a “disproportionate” FMR with a RF of 50%, a LVEDV of 160 ml and a LVEF of 30% LVSV_tot_ is 48 ml and LVSV_eff_ is 24 ml. Thus, a heart rate of 135/min is needed to obtain a CI of 1.7 l/min/m^2^ (1700 ml x 1.9 m^2^/24 ml), and a heart rate of 158/min to ensure a CI of 2.0 l/min/m^2^ (2000 ml x 1.9 m^2^ / 24 ml), respectively.

In conclusion, hemodynamics should be critically discussed and pose the question “whether hemodynamic considerations should be checked with respect to plausibility and should be integrated into the approach of assessing MR severity or not?” (6).

## Reasons for Echocardiographic Results Describing a Disproportionateness Between EROA and LVEDV

“Disproportionate” FMR describes the disproportionateness between EROA and LVEDV (12–14). However, in MR patients—especially in FMR patients—LVEDV can only be interpreted if LV function will be considered described by LVEF (6, 15–17). In isolated MR, LVSV_tot_ can be determined by the addition of LVSV_eff_ and the transmittal regurgitant volume (MR_RegVol_) during systole provided that LVEDV and LVEF are properly assessed. According to physical laws in communicating tubes proportionality always exists between volume flow and cross-sectional area (CSA) or between flow velocities and CSA. In echocardiography, it must be considered that spectral and color-coded Doppler signals represent blood flow velocities, not volume flow.

The proportionality between flow velocities and CSA are accepted for the estimation of the effective orifice area (EOA) in aortic valve stenosis (AS) by the continuity equation (1, 3, 5). Because of physical laws of conservation of mass and energy in communicating tubes the same physical principle applies to EROA and MR_RegVol_ in MR (6). Thus, the EROA should theoretically be calculated by the equation CSA_MA_ x VTI_MAret_ = EROA x VTI_MRret_, where CSA_MA_ is the cross-sectional area of the mitral annulus (MA), VTI_MAret_ is the velocity time integral of the systolic retrograde flow velocities determined at the MA level by pw Doppler and VTI_MRret_ is velocity time integral of the systolic retrograde transmittal flow velocities determined by cw Doppler. However, it is not possible to perform these measurements to analyze EROA in MR patients like in AS patients due to the deceleration of retrograde transmittal flow velocities and the impossibility to assess high regurgitant flow velocities at the mitral annulus (MA) level by pw Doppler (6, 16). However, due to physical laws of conservation of mass and energy in communicating tubes, disproportionateness between EROA and LVEDV is impossible provided that LV function is properly assessed. If disproportionateness results from echocardiographic calculations, measuring errors must be assumed as the only explanation. In addition, or with other words, it is a fact that the more “disproportionate” the MR, the more measuring errors are the reason for “disproportionate” MR (16).

With respect to the methodological challenges, three main reasons of measuring errors for the assessment of MR severity by echocardiography must be discussed: (1) the assessment of LV volumes (18), (2) the assessment of MR_RegVol_ by the 2D-PISA (proximal isovelocity surface area) method (2, 4, 6)—and (3) the time point and the conditions of baseline echocardiography to characterize the hemodynamic state of MR prior to any therapy (6).

### The Assessment of LV Volumes

**Figure 1:**
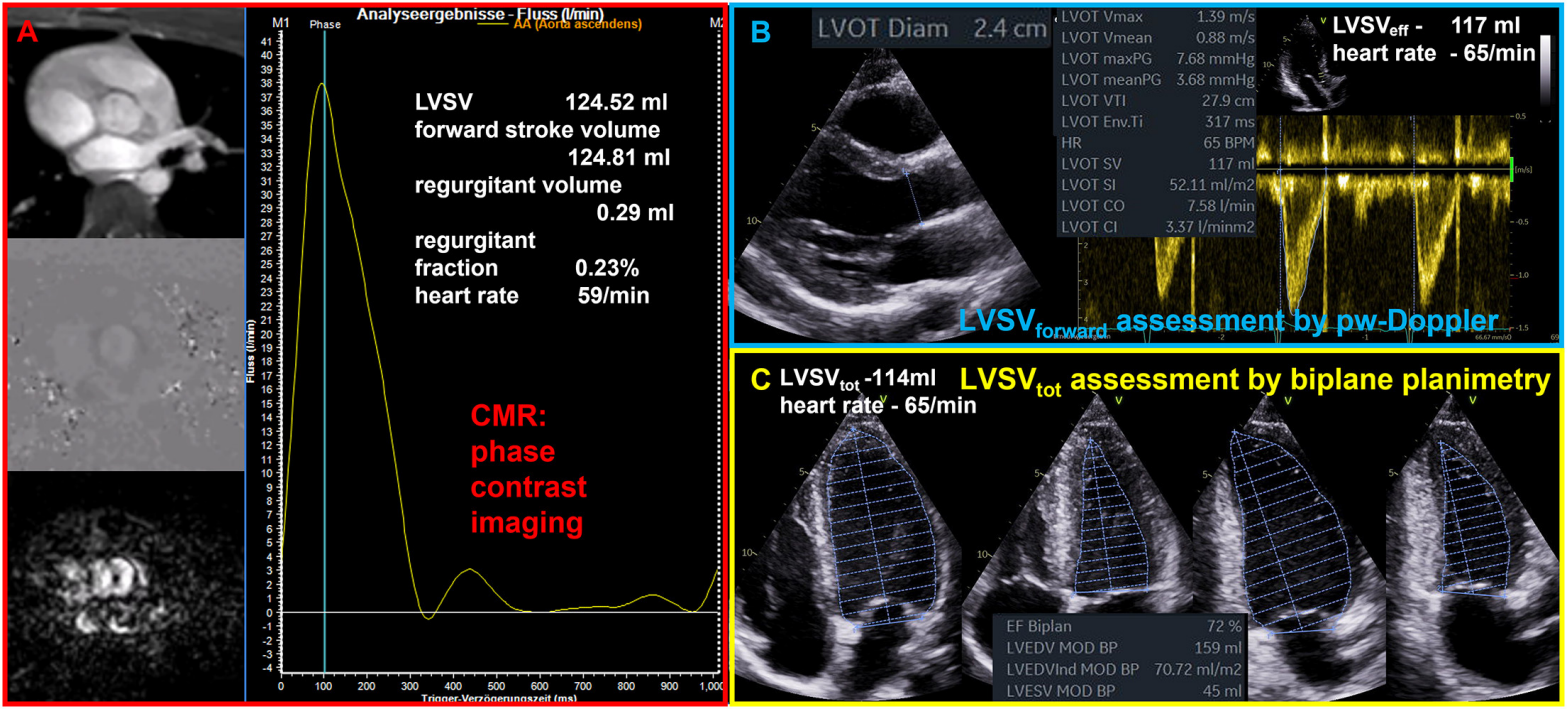
Illustration of comparable quantitative assessment of left ventricular stroke volume (LVSV) by cardiac magnetic resonance tomography (CMR) and echocardiography. **(A)** Phase contrast imaging and assessment of forward flow through the aortic valve by CMR phase contrast imaging. **(B)** Assessment of forward LVSV through the aortic valve by pulsed wave (pw) Doppler (LVSV_forward_); LVSV_forward_ is the effective LVSV (LVSV_eff_) as well as the total LVSV (LVSV_tot_) during normal conditions. **(C)** Assessment of LVSV_tot_ by biplane planimetry.

**Figure 2:**
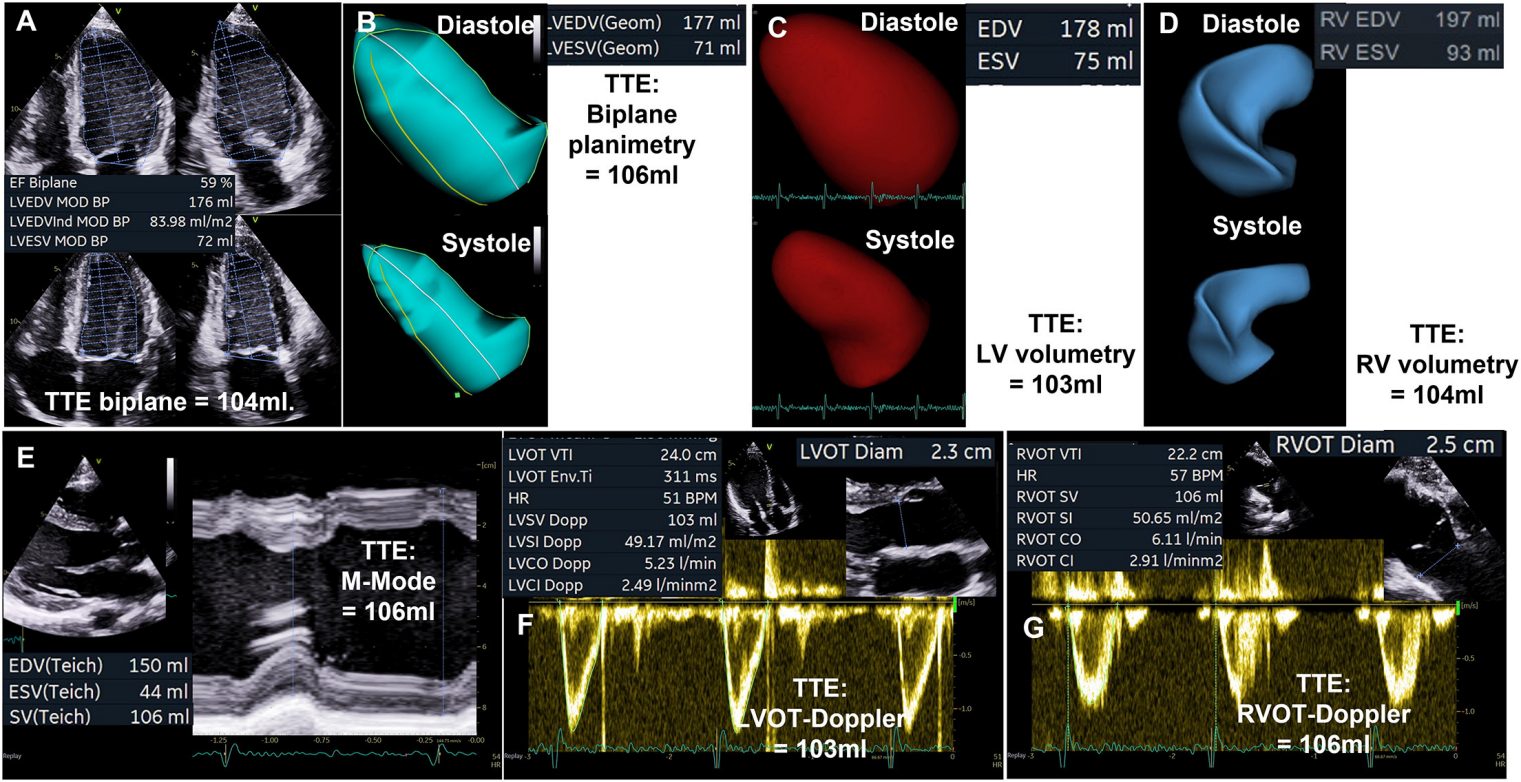
Illustration of comparable quantitative assessment of left ventricular stroke volume (LVSV) by different echocardiographic modalities. **(A)** Biplane planimetry of the left ventricle (LV). **(B)** Triplane LV planimetry. **(C)** LV volumetry by 3D transthoracic echocardiography (TTE). **(D)** Volumetry of the right ventricle (RV) by 3D-TTE. **(E)** LVSV determination by M-Mode analysis. **(F)** LVSV assessment by measurement of blood flow velocities in the left ventricular outflow tract (LVOT). **(G)** RV stroke volume (RVSV) assessment by measurement of blood flow velocities in the right ventricular outflow tract (RVOT).

The quantitative assessment of LV volumes (LVEDV and LV end systolic volume - LVESV) by TTE—especially in comparison to cardiac magnetic resonance tomography (CMR)—is an ongoing debate (19, 20). A significant underestimation of LVEDV by native 2D echocardiography is often reported compared to CMR, which is considered as the gold standard of cardiac volume assessment. Comparable or even overestimated LV volumes determined by native 2D echocardiography in comparison to CMR were only reported in a minority of studies (19). LV volume differences between both modalities are not induced by the modalities *per se*, but rather by the consequences of methodological inaccuracies. Thus, equivalent approaches should be compared, such as biplane measurements by TTE and CMR, or short axis LV assessment by respective sectional slices within a 3D-TTE data set and CMR short axis packages. Whereas, the main echocardiographic problem is the delineation of the LV endocardium including the trabecula into the LV cavity (19), there are other reasons for measuring larger LV volumes by CMR such as different orientation of 2- and 4-chamber view (2, 4 ChV) in comparison to TTE (in CMR studies 2 ChV is often perpendicular to the 4 ChV instead of a 60° rotation difference in TTE studies.) and lower heart rates in CMR than in the comparable TTE documentation at rest. The comparability of LVSV_eff_ measurements between echocardiography and CMR (see Figure 1) as well as between echocardiographic modalities (see Figure 2) are illustrated during normal conditions. With respect to the accurate cardiac volume assessment proposed in patients with valvular regurgitations by recent recommendations (4), approaches performed by CMR can comparably be performed by echocardiography providing a high level of standardization and image quality.

### The Assessment of MR_RegVol_ and EROA by the 2D-PISA Method

Although it is not recommended to use the jet area for MR quantification and despite of the inappropriateness to quantify MR severity by its size and its relation to the left atrium, this approach is still the most often so-called semi quantitative parameter that is used for grading of MR severity (21). The second most common method is the 2D-PISA method with its well-known limitations (21). It is obvious that dynamics of MR during systole restrict this method to a special—presumably small—subgroup of MR or FMR-patients, in whom the PISA radius is flat, and in whom the jet direction is central and perpendicular to the MA. However, such as in COAPT more than 90% of the enrolled patients were classified by the 2D-PISA method (22).

**Figure 3:**
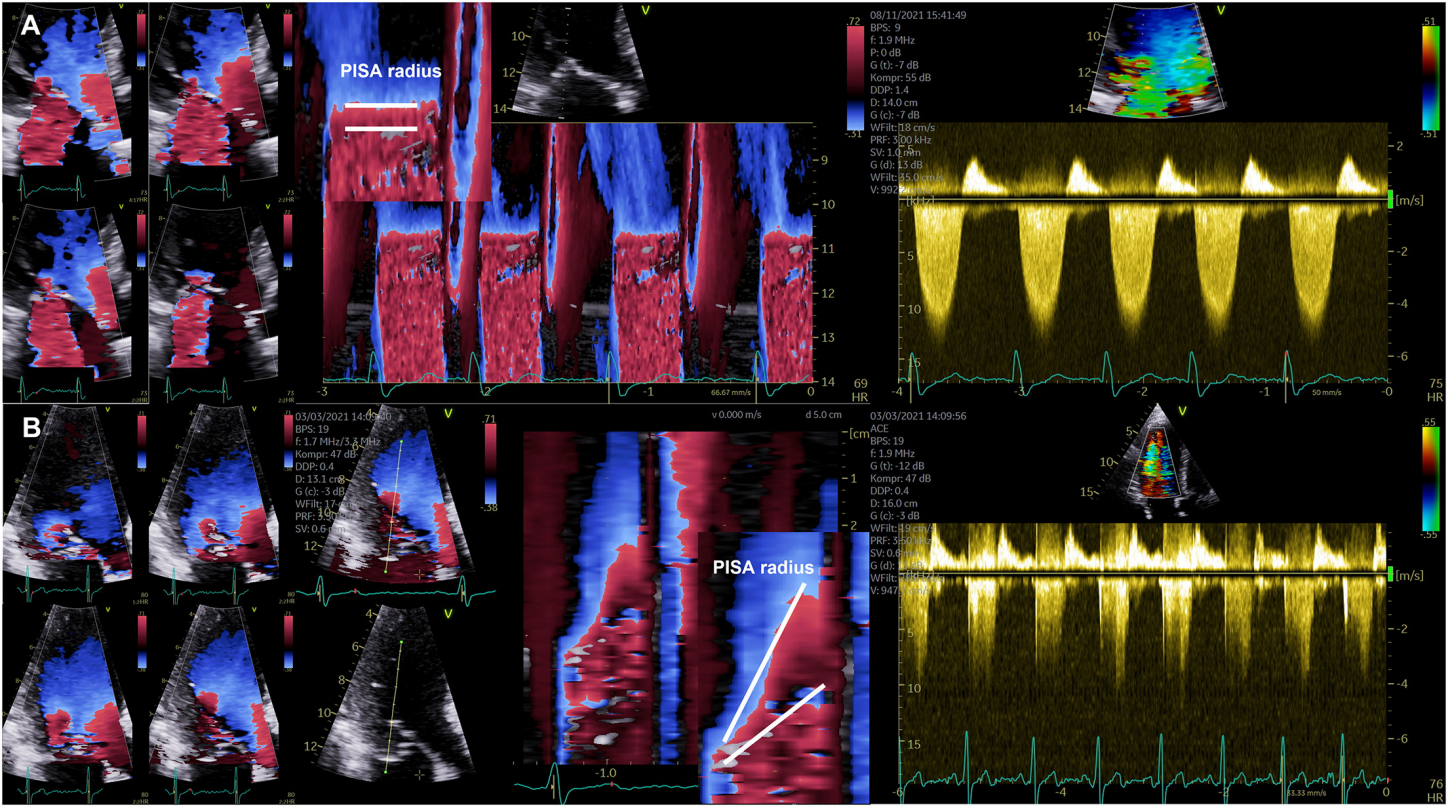
Illustration of the dynamics of the PISA radius in mitral regurgitation (MR). **(A)** Documentation of almost stable PISA radius in a patient with secondary MR. On the left side four consecutive images during systole are shown illustrating the almost constant PISA radius. Right to these images a color-coded M-Mode illustrating the horizontal PISA radius and the continuous wave Doppler spectrum are shown. **(B)** Documentation of severe late systolic increase of PISA radius in a patient with primary MR. On the left side four consecutive images during systole are shown illustrating the significant increase of PISA radius at late systole. Right to these images a color-coded M-Mode illustrating the dynamics of the PISA radius and the continuous wave Doppler spectrum with the late time-to-peak regurgitant flow velocities are shown.

Both, EOA in AS, and EROA in MR are functional parameters, which reflect the functional EOA or EROA during systole. However, due to the dynamics of MR the EROA measured by the 2D-PISA does usually not reflect the functional EROA during systole. Due to the limitations of the 2D-PISA method the term EROA should be better labeled as EROA_by 2D−PISA_ to avoid misunderstanding and misinterpretation (4, 6). The proper labeling of the origin of the 2D-PISA radius—entry of the orifice area vs. vena contracta—(4, 6) is one crucial point, the proper imaging of the convergence area, MR dynamics within the cardiac cycle, and ultrasound settings are additional sources of errors. In most of the cases the assessment of the EROA_by 2D−PISA_ is the so-called maximum EROA, in which the 2D-PISA method is not applicble. An appropriate use of the 2D-PISA method can be verified by a homogenous PISA radius in the color-coded M-mode and by the ability to delineate the regurgitant velocity contour in the cw Doppler spectrum (see Figure 3).

**Figure 4:**
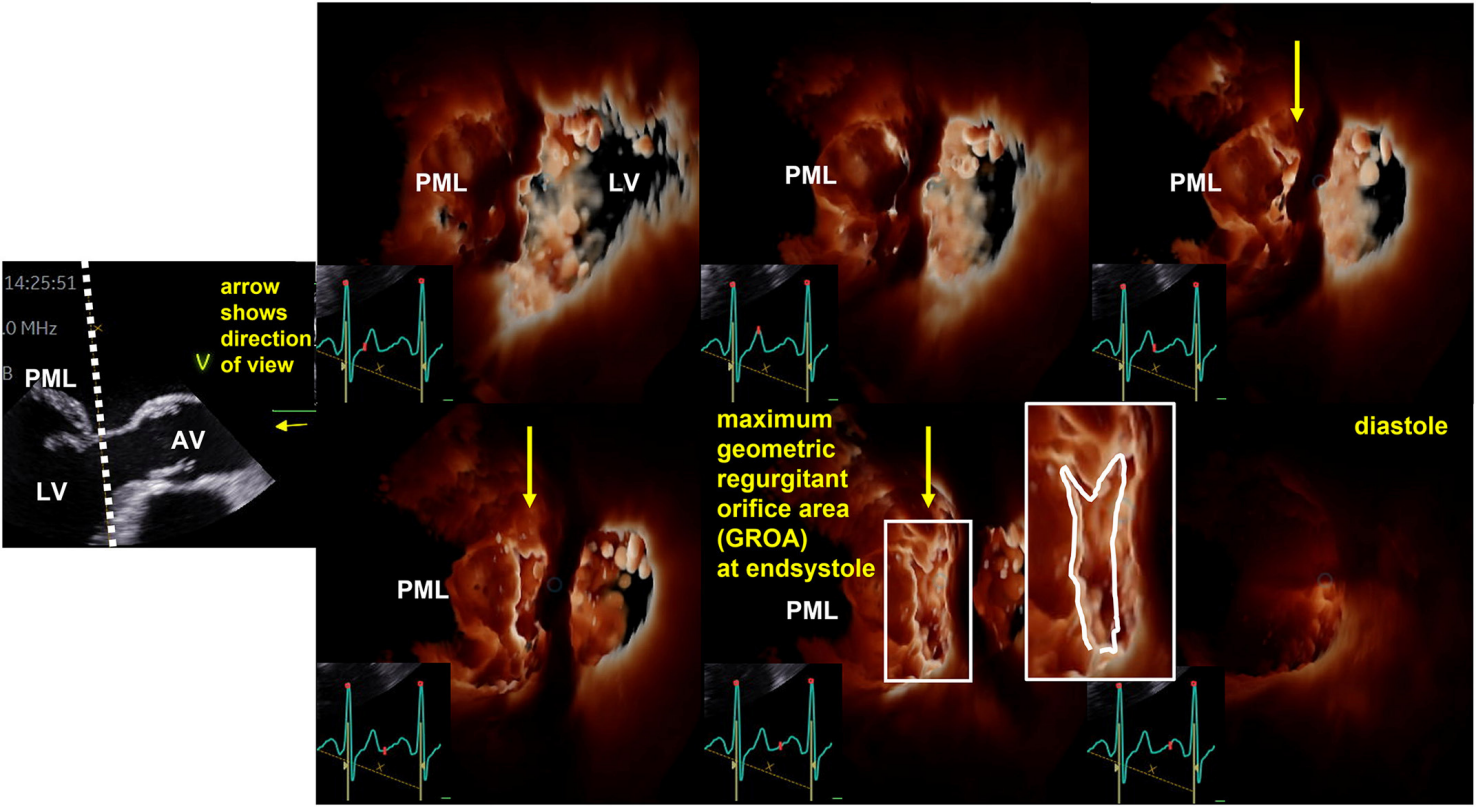
Illustration of the dynamics of geometric regurgitant orifice area (GROA) in a patient with primary mitral regurgitation by 3D transesophageal echocardiography (TEE). On the left side the sectional plane of a TEE long axis view is shown. The dotted line represents the cutting plane of the en-face view to the GROA. The direction of view is illustrated by the yellow arrow. AV, aortic valve; LV, left ventricle; PML, posterior mitral leaflef. Right to this 2D image six consecutive 3D-en-face views to the GROA are presented illustrating the maximum GROA at end systole.

The CSA of the left ventricular outflow tract (LVOT) is relatively stable and transvalvular flow velocities are integrated during systole, which is why the EOA of AS can be assumed as a relatively stable value, if certain measuring errors will be avoided. In contrast—especially in eccentric MR—the velocity time integral (VTI) of regurgitant flow velocities is difficult to delineate and the PISA-radius using the 2D-PISA method varies during systole. In primary MR often a late systolic regurgitation with a late time-to-peak-shape can be observed. In FMR the PISA-radius also varies during systole in many cases. Thus, variations of both parameters cause a wide range of measuring errors often resulting in overestimation of MR_RegVol_ and EROA with incongruences of echocardiographic measurements. The problem of assessing EROA by EROA_by 2D−PISA_ leads to many different terms, e.g., mean or maximum EROA. EROA_by 2D−PISA_ is also interpreted as a substitute of the mean or maximum geometric regurgitant orifice area (GROA) (22–24). The differences of GROA can be assessed and visualized by 3D-TEE indicating relevant MR dynamics in MR patients, in whom the 2D-PISA is inappropriate (see Figure 4).

In isolated FMR systolic regurgitant transmittal flow (MR_RegVol_) can be determined by the difference of LVSV_tot_ and LVSV_eff_. Consecutively, EROA can be calculated by the equation MR_RegVol_/ VTI_MR_, whereas MR_RegVol_ is defined as LVSV_tot_ - LVSV_eff_, and not MR_RegVol_ determined by 2D-PISA. However, the quantitative assessment of LVSV_tot_, LVSV_eff_, and MR_RegVol_ requires a highly standardized and comprehensive echocardiography to ensure reliable, verifiable, and reproducible data (6). Considering this approach the calculated EROAs determined in recent TMVR trials are <0.2 cm^2^ representing conditions of less than severe FMR (15–17, 25).

### The Time Point and the Conditions of Baseline Echocardiography to Quantify MR Severity

Beside the dynamic variations of GROA during systole, the degree of MR—especially of FMR—is dynamic and depends on several aspects. Thus, the circumstances to compare FMR severity at two time points, which should be representative for alterations of FMR severity—need to be defined exactly. Thus, parameters such as body weight, blood pressure, heart rate, intravascular volume conditions and drug treatment should be within comparable ranges during both echocardiographic investigations. It was recently proposed to perform baseline TTE one day prior to intervention or surgery during real-life resting conditions with OMT (6). This baseline TTE should be compared to the TTE at early hospital discharge. To analyze the acute effect of intervention baseline TEE should be performed directly prior to device employment during the monitoring procedure, and the FMR status at this baseline TEE should be compared with the status directly after device employment (6). It is obvious that the conditions of a baseline TEE in left lateral position prior to the intervention without sedation are different to the conditions at intervention during anesthesia. In recent TMVR trials the degree of baseline MR severity directly prior to intervention with OMT remains uncertain, because the time interval between baseline TTE and intervention is not clearly defined (e.g., in COAPT baseline TTE was within 90 days, baseline TEE within 180 days prior to the intervention) (9–11). In addition, medical therapy differed between treatment and control group which might lead to misinterpretation of post-procedural effects (26, 27).

## The Discussion About the Treatment Target of TEER Therapy

Although the characterization of MR severity by echocardiographic estimations in recent TMVR trials is debatable because of obviously not possible hemodynamic conditions (9–11), the randomized prospective COAPT trial documented a significant treatment effect favoring the device therapy (10), whereas no effect was described in the MITRA-FR trial (9). These different results initiated a still ongoing debate about the differences between the echocardiographic characteristics in both trials (12–17, 28–30) creating the terms “proportionate” and “disproportionate” FMR as “a new conceptual framework that reconciles the results of the MITRA-FR and COAPT trials” (12). There are at least two explanations for the created new term “disproportionate” FMR. First of all, it might be assumed, that the hemodynamic disproportionality between orifice areas and flow velocities based on inconsistent measurements describes a different pathophysiological condition than the MR severity in relation to LV size. Probably TEER therapy might address HF therapy independently of MR severity. Of course, FMR is frequently observed in many HF patients, and the more severe the FMR, the more frequent are clinical complaints of HF.

Assuming that MR severity was not correctly assessed in recent TMVR trials, and that MR severity was less than severe and matched according to Gaussian distribution with unknown maximum peak, it can be concluded, that another aspect must be the reason for the treatment success in CPAT but not severe FMR. It is often discussed that TMVR and surgical FMR therapy fails if therapy comes too late. The underlying pathophysiology of this scenario might be the progression of LV dysfunction due to the missing LV capability of reverse remodeling. When the LV is fully enlarged as a result of severe FMR, LV reverse remodeling is presumably not possible anymore. Thus, the treatment effect of TEER might be the prevention of repetitive LV decompensation in HF patients regardless of MR severity. With other words, if HF patients are at high risk of LV decompensation under OMT, an early intervention might be helpful regardless of MR severity. This is supported by the fact MR severity has been overestimated in all TMVR trials, if MR_RegVol_-calculations are adjusted to hemodynamic conclusiveness (16). However, this hypothesis about HF patients with mild and moderate FMR needs to be verified by further studies. The uncertainty of FMR severity and the presumably significant overestimation of FMR severity in COAPT indirectly support the hypothesis that the capacity of LV reverse remodeling is the real cause of the treatment success in COAPT. Thus, the chance of LV reverse remodeling might be better, if LV is less remodeled, which is in line with less dilated left ventricles (9, 10).

## The Dilemma of Inconsistent Functional Echocardiographic Data

A common argumentation to assess MR severity by the 2D-PISA method despite its limitations is the fact that data derived by 2D-PISA can be used to estimate prognosis in patients with MR (14, 18). There are several trials confirming that the more severe the MR, the worse the patient's prognosis (31), which is not surprising, because the more the pathology is apart from normal, the worse the expected prognosis.

However, there are some crucial points regarding this argumentation with respect to individual decision making (32–34). Considering epidemiological aspects, the ranges of MR severity are only reliable to distinguish between mild, moderate, and severe MR, if everyone will make the same measuring errors, which presumably does not reflect reality. Thus, despite an acceptable epidemiological approach for grading of MR severity using group comparisons, the 2D-PISA method is not suitable for individual estimation of MR severity. Further, it is difficult to interpret data, which document a higher MR_RegVol_ compared to LVSV_eff_, which is obviously not possible (10), or hemodynamic conditions, that require heart rates of more than 100/min to ensure a CI of at least 1.7 l/min/m^2^ (9), which is obviously not in line with OMT. It must be fairly conceded, that if calculations document implausible hemodynamics, any degree of MR severity can be assumed depending on the extent of the measuring errors. Thus, results describing a so-called “disproportionate” FMR are misleading and cannot be the base of reliable interpretations. In addition, a proper individual decision making on the basis of inconsistent data is impossible. In consequence, plausible estimations of LVSV_tot_, LVSV_eff_, MR_RegVol_, and RF are necessary to assess the individual MR severity (6). Finally, if categorization of MR severity is based on an inconclusive assessment by the PISA method, the limits of severe MR (MR_RegVol_ > 60 ml and RF > 50%) as presented in recent recommendations (2, 4, 32–34) must be reevaluated and redefined based on conclusive hemodynamics.

Plausible functional diagnostics by rational echocardiography probably can untangle the Gordian knot of echocardiographic MR assessment (17). Thus, rational echocardiography is based on thorough methodology and correct measurements to enable appropriate conclusions. Nevertheless, echocardiography is sometimes limited by subjectivity and high interobserver. These limitations must be minimized by performing comprehensive echocardiography and by improving methodological accuracy. The plausibility is the second pillar of rational echocardiography. Plausible echocardiographic results should be conclusive and transparent implying a verifiable documentation. Thus, the exclusion principle can be used to reveal wrong results owing to methodological absurdity (such as retrograde arterial blood flow due to MR). On the other hand, a functional cardiac scenario can be confirmed by a logical causation. In conclusion, the aim of functional diagnostics by rational echocardiography is the thorough characterizations of cardiac physiology and pathophysiology. Echocardiographic estimations based on hemodynamic plausibility can be considered as a quality check of echocardiographic documentation and measurements (6).

If echocardiographic results seem to be inappropriate to correctly characterize hemodynamics, the severity of valvular defects remains unclear. Misinterpretation of the results might have serious consequences for individual decision making and interpretation of scientific results.

FMR severity can correctly be assessed by plausible echocardiography if comparable hemodynamics are assumed in patients with implemented OMT, especially when the treatment success of TMVR needs to be investigated in the context of clinical trials. The assessment of FMR severity at a so-called baseline echocardiography in patients with not-optimized medical treatment defines an unrepresentative scenario which should not be the basis for discussions in the heart team. In consequence with respect to clinical routine,—if surgery will be recommended—the patient will be reevaluated by echocardiography prior to surgery at hospital admission several days later. If clinical improvement will be observed in the meantime documented by mild to less than severe FMR surgery would presumably be canceled. The reason for the observed clinical improvement might presumably be a delayed response to OMT or an intensification of OMT due to better hemodynamics documenting that OMT did not respond at the timepoint of baseline echocardiography. If the decision is made for interventional therapy the patient will often be treated earlier—sometimes during the same hospitalization without performing another echocardiography prior to the intervention. Thus, the detection of potential changes of FMR severity with time would fail if verifiable echocardiographic investigations are not repeated within comparable pre-interventional time periods. The results of potential different study designs might create uncertainty to allocate the underlying treatment effects. What needs to be done to improve diagnostics to ensure comparable baseline conditions?

1. The screening of FMR patients according to jet area without hemodynamic quantification during non-defined conditions is misleading. FMR patients should be selected using a specific protocol and under stable conditions with OMT at baseline echocardiography. The verifiable quantitative baseline echocardiography should be repeated after a representative time interval prior to the intervention to exclude hemodynamic changes during OMT and to verifiy the treatment effects of OMT.
2. Due to the uncertainty of FMR severity in recent TMVR trials baseline echocardiography directly prior to the intervention should document comparable hemodynamics with the screening TTE and TEE with OMT to allocate conclusively the interventional treatment effect.
3. The cohorts of patients treated conventionally, or by surgery or by TMVR should provide proven comparable FMR severity with OMT at baseline in clinical trials. Considering the long time interval of baseline echocardiography prior to inclusion and the documented differences of drug treatment between the cohorts after intervention (10) the causal relationship of treatment effects can still be discussed.

Thus, the current concepts of FMR quantification require validation preferably by data based on verifiable and transparent assessment of FMR severity by echocardiography (16, 24). Comparable to CMR, echocardiography enables a quantitative assessment of cardiovascular hemodynamics. Thus, comprehensive echocardiography must provide verifiable data to clarify the objectives of treatment strategies in FMR patients. Up to the present, the *post-hoc* analyses of inconclusive echocardiographic data (12–14, 22) and the creation of new parameters and ratios, characterizing FMR severity (29, 30) may presumably not contribute to the solution of the ongoing debate.

## Summary and Conclusions

Plausible functional diagnostics by echocardiography enables a conclusive and quantitative analysis of hemodynamics in VHD. The integrated approach to grade MR severity by semi quantitative parameters is often misleading. The main sources of errors by echocardiography include underestimation of LV volumes—mainly due to foreshortening and non-standardized views—and overestimation of regurgitant volumes and regurgitant fractions calculated by the 2D-PISA method due to several limitations. The hemodynamic assessment of FMR severity must be performed under comparable circumstances with OMT. Investigational circumstances must distinguish between life conditions the day prior to and after the intervention by TTE, and sedated conditions directly prior to and after device deployment by TEE. In all recent TMVR trials FMR severity can be assumed to be less than severe according to hemodynamics. Thus, due to the uncertainty of FMR severity the target of treatment remains unclear. The prevention of future LV decompensation after TEER treatment might by a more important issue than the treatment of a severe FMR. This hypothesis sets up the stage for further studies to determine the prognostic value of TMVR treatment.

## Data Availability Statement

The original contributions presented in the study are included in the article/supplementary material, further inquiries can be directed to the corresponding author/s.

## Author Contributions

Both authors listed have made a substantial, direct, and intellectual contribution to the work and approved it for publication.

## Conflict of Interest

The authors declare that the research was conducted in the absence of any commercial or financial relationships that could be construed as a potential conflict of interest.

## Publisher's Note

All claims expressed in this article are solely those of the authors and do not necessarily represent those of their affiliated organizations, or those of the publisher, the editors and the reviewers. Any product that may be evaluated in this article, or claim that may be made by its manufacturer, is not guaranteed or endorsed by the publisher.
